# Comparison of hemagglutination inhibition, single radial hemolysis, virus neutralization assays, and ELISA to detect antibody levels against seasonal influenza viruses

**DOI:** 10.1111/irv.12591

**Published:** 2018-08-11

**Authors:** Claudia Maria Trombetta, Edmond J. Remarque, Daniella Mortier, Emanuele Montomoli

**Affiliations:** ^1^ Department of Molecular and Developmental Medicine University of Siena Siena Italy; ^2^ Department of Virology Biomedical Primate Research Centre Rijswijk The Netherlands; ^3^ VisMederi srl Siena Italy

**Keywords:** correlates of protection, ELISA, hemagglutination inhibition assay, single radial hemolysis, virus neutralization assay

## Abstract

**Background:**

The immunological response to influenza vaccine and/or natural infection is evaluated by serological techniques, the most common being hemagglutination inhibition (HI), single radial hemolysis (SRH), and virus neutralization assays, which is commonly used in a micro‐neutralization (MN) format. ELISA is not officially required; however, this assay is able to measure different class‐specific antibodies. The four assays identify different sets or subsets of antibodies.

**Objectives:**

The aim of this study was to establish the correlation among four serological assays using four seasonal influenza strains.

**Methods:**

The HI, SRH, MN assays, and ELISA were performed on four seasonal influenza strains.

**Results:**

A strong positive correlation was found between HI and MN and between SRH and MN assays for influenza A strains. The B strains also showed good correlations among the three assays. A positive correlation was also found between ELISA and the “classical” assays for all strains. Concerning the correlates of protection, as defined by HI ≥ 40 and SRH ≥ 25 mm^2^, good agreement was observed for the influenza A strains. By contrast, the agreement for the B strains was very low.

**Conclusions:**

There is a positive strong correlation among the four serological assays for both A and B strains, especially for the HI and MN assays. There is good agreement on correlates of protection between HI and SRH assays for the A strains, but very low agreement for the B strains, suggesting higher sensitivity of SRH than HI assay in detecting antibodies against the influenza B viruses.

## 1 INTRODUCTION

The immunological response to influenza vaccine and/or natural infection is evaluated by serological techniques recommended by regulatory authorities. The most common and widely used are the hemagglutination inhibition (HI) and the single radial hemolysis (SRH) assays, which are officially recognized by the European Medicines Agency (EMA).^1^

The HI assay is considered the gold standard as a correlate of protection for influenza vaccines^2^, ^3^, ^4^ and has proved to be simple, rapid, and cost‐effective. The aim of the assay is to detect antibodies, capable of inhibiting the agglutination between red blood cells and the viral hemagglutinin (HA).^5^ The HI titer is expressed as the reciprocal of the highest serum dilution that shows complete inhibition of agglutination.^6^ An antibody titer of 40 is generally regarded as a protective threshold level, beyond which there is less than 50% chance of contracting influenza infection.^7^

Despite its wide application, the assay has limitations, including low sensitivity for influenza B and avian viruses, inadequacy in the evaluation of live attenuated vaccines and a high degree of variability among laboratories, due to many factors, including the source of reagents (such as red blood cells and receptor‐destroying enzyme) and the lack of standardized protocols.^7^, ^8^, ^9^

The SRH assay is a robust technique based on the passive hemolysis of red blood cells, which is mediated by complement and induced by the antibody‐antigen complex. The hemolysis produces an easily identifiable “area of hemolysis,” which is proportional to the concentration of influenza antibodies, mainly IgG, present in serum samples.^3^, ^10^, ^11^ The advantages of the SRH assay are the small quantities of influenza virus and serum required, the ability to simultaneously analyze a large number of serum samples without pre‐treatment (apart from complement inactivation) and unbiased results available after overnight incubation.^12^ In addition, the assay detects small differences in antibody levels, distinguishes differentiates between closely related influenza strains, and is more sensitive for influenza B strains than the HI assay.^12^, ^13^, ^14^, ^15^ A hemolysis area of 25 mm^2^ or greater is generally considered to be an immunological correlate of protection.^16^

Another widely used serological technique is the virus neutralization assay, which is recommended by the World Health Organization (WHO) for the measurement of functional antibodies against highly pathogenic avian viruses^6^ and currently included in the EMA guidelines on influenza vaccines.^1^ Commonly used in a micro‐neutralization (MN) format, this assay detects antibodies at low titers and distinguishes between pre‐ and post‐vaccination titers in paired sera, especially in the case of small (less than 2‐fold) differences in titers.^17^ The disadvantage lies in the handling of wild‐type viruses, which, in the case of highly pathogenic strains, require high‐level facilities. The assay suffers from high interlaboratory variability, owing to the lack of common reference protocols and discrepancies in endpoint determination. Here, MN titer is expressed as the reciprocal of the serum dilution showing at least 50% inhibition of cytopathic effect in mammalian cell culture.^7^, ^18^ To date, no correlates of protection have been established for the MN assay.

In addition to the traditional immunological techniques, the enzyme‐linked immunosorbent assay (ELISA) detects influenza antibodies. Advantages are its ability to measure different class‐specific IgM, IgA, and IgG antibodies in serum samples and nasal wash in response to influenza infection and/or vaccine and to use a wide range of antigen preparations. This assay is particularly suitable for large‐scale serological investigations, as it yields unbiased results in a few hours and is cost‐effective and amenable to complete automation. In addition, the ELISA assay is reproducible and reagents can be standardized (e.g, coating antigen, conjugate for Ig detection).^7^, ^19^, ^20^ ELISA mainly detects anti‐HA antibodies, even when the whole virus is used, as HA is immunodominant.^21^, ^22^ ELISA can also be used to detect responses to other influenza antigens when purified single antigens are used for coating. The use of purified HA antigens or a recombinant fragment of the HA globular head may considerably improve the specificity of ELISA.^7^, ^19^, ^23^, ^24^, ^25^, ^26^

The four assays identify different sets or subsets of antibodies. The HI assay detects antibodies that bind to the viral HA and prevent the virus‐red blood cells agglutination by blocking the receptor binding site; the MN assay identifies functional neutralizing antibodies, including those that recognize epitopes in the stem region of HA, which are conserved among different subtypes of influenza A viruses.^17^, ^20^, ^27^ Consequently, the MN assay could be less specific in adults and elderly subjects with extensive previous exposure to influenza viruses.^27^ However, MN is more sensitive than HI, particularly in detecting low‐titer seroconversions. A combination of assays could improve the sensitivity and specificity of influenza vaccine evaluation.

The SRH assay may recognize antibodies not only against the surface glycoproteins but also those against the internal antigens, leading to a potential lack of specificity to antibodies against HA.^3^ However, the immune response to HA is immunodominant over the response to other viral proteins, such as neuraminidase and internal proteins.^21^

Several studies have compared serological techniques for the evaluation of antibody response to influenza viruses or vaccination. Overall, the HI, SRH, and MN assays have shown significant positive correlations, especially for influenza A strains.^11^, ^14^, ^27^, ^28^, ^29^ A recent study has revealed that the correlation between SRH and MN is greater than that between SRH‐HI and HI‐MN assays.^30^ ELISA shows good agreement between the HI and MN assays; notably, ELISA seems to be more sensitive than HI assay, especially in detecting low levels of antibody, owing to the low background.^24^, ^31^, ^32^

Few previous studies have dealt with the correlations among the four assays.

The aim of this study was to establish the correlations among the four immunological assays: HI, SRH, MN, and ELISA using four egg‐grown seasonal influenza strains.

## 2 MATERIALS AND METHODS

### 2.1 Virus antigens

The virus antigens and infectious influenza viruses were seasonal influenza strains obtained from NIBSC: A/California/7/2009 (H1N1, 12/168‐15/252), A/Texas/50/2012 (H3N2, 13/112‐12/298), B/Brisbane/60/2008 (B, 13/234‐15/146) (Victoria lineage), and B/Massachusetts/02/2012 (B, 13/106‐14/106) (Yamagata lineage). Live viruses were used for MN assay; inactivated antigen preparations, obtained from NIBSC, were used for HI, SRH, and ELISA. All viruses used were egg‐grown.

### 2.2 Serum samples

Human serum samples (n = 450) from adults were obtained from the Serum Bank of the Laboratory of Molecular Epidemiology, Department of Molecular and Developmental Medicine, University of Siena, Italy. The samples were collected anonymously and stored in compliance with Italian ethics law.

The only information available for each serum sample was the subject's age and the year of sampling. Serum samples were randomly selected from a total of 600 samples available at the Serum Bank and collected in the area of Siena in 2015. The selection was performed by means of a randomization list, the main selection criterion being balanced numbers for gender and age.

Influenza sheep hyperimmune serum samples were provided by NIBSC and were used as positive controls: A/California/7/2009 (H1N1, 09/152), A/Texas/50/2012 (H3N2, 13/110), B/Brisbane/60/2008 (B, 11/136), and B/Massachusetts/02/2012 (B, 13/182).

Human serum without IgA, IgM, and IgG was used as negative controls (Sigma‐Aldrich, S5393).

### 2.3 Hemagglutination inhibition assay

All serum samples, including the sheep serum samples and negative control, were pre‐treated with receptor‐destroying enzyme (RDE) (ratio 1:5) from Vibrio Cholerae (Sigma‐Aldrich, Milan, Italy) for 18 hours at 37°C in a water bath and then heat‐inactivated for 1 hour at 56°C in a water bath with 8% sodium citrate (ratio 1:4).

Fresh turkey red blood cells were centrifuged twice, washed with a saline solution (0.9%), and adjusted to a final dilution of 0.35%.

From an initial dilution of 1:10, serum samples were 2‐fold diluted in duplicate with saline solution (0.9%) in a 96‐well plate; 25 μL of standardized viral antigen (4 HA units/25 μL) was added to each well, and the mixture was incubated at room temperature for 1 hour. Red blood cells were added and, after 1‐hour incubation at room temperature, the plates were evaluated for the presence of agglutination inhibition.

The antibody titer was expressed as the reciprocal highest serum dilution that showed complete inhibition of agglutination. As the starting dilution was 1:10, the lower limit of the detectable antibody titer was 10. When the titer was below the detectable threshold, the results were conventionally expressed as 5 for calculation purposes, half the lowest detection threshold.

### 2.4 Single radial hemolysis assay

Before being used in the SRH assay, serum samples were heat‐inactivated at 56°C for 30 minutes in a water bath. Fresh turkey red blood cells were centrifuged and washed twice with phosphate buffer saline (PBS). Diluted virus antigen was added to the red blood cell suspension at a concentration of 2000 hemagglutinin units (HAU) per milliliters (mL). In order to allow the adsorption of viral antigen to the red blood cells, the suspension was incubated at 4°C for 20 minutes. A solution of chromium chloride (CrCl_3_) 2.5 mmol/L was added to the suspension and incubated at room temperature for 10 minutes to increase the binding affinity between the red blood cells and the viral antigen. The suspension was mixed once and subsequently centrifuged. The supernatant was removed, PBS was added, and the pellet was carefully re‐suspended. A stock solution of 1.5% agarose‐agarose low gelling in PBS containing 0.1% sodium azide was prepared. The agarose stock solution was kept at 45°C in a water bath.

Each SRH plate contained red blood cells‐viral antigen suspension and guinea pig complement in the agarose mixture. The final suspension was vigorously shaken and then evenly spread onto each plate and incubated at room temperature for 30 minutes, and the agarose was allowed to set at 4°C for 30‐90 minutes. Holes were made in each plate with a calibrated punch, and 6 microliters (μL) of serum samples and controls was added through each hole. The plates were stored in a humid box and incubated at 4°C for 16‐18 hours in the dark. After overnight incubation, the plates were incubated in a water bath at 37°C for 90 minutes, after which the diameters of the hemolysis areas were read in millimeters (mm) with a calibrated viewer.^12^

### 2.5 Micro‐neutralization assay

Madin Darby Canine Kidney (MDCK) cells were maintained for a maximum of 30 passages in EME medium containing 10% fetal bovine serum (FBS), 2 mmol/L L‐glutamine, 1% non‐essential amino acid solution, and 100 U/mL penicillin‐streptomycin. The MDCK cell cultures were grown at 37°C in 5% CO_2_. Serum samples, previously heat‐inactivated at 56°C for 30 minutes, were diluted 2‐fold with EMEM culture medium supplemented with 0.5% FBS in a 96‐well plate, mixed with an equal volume of virus (100 TCID_50_/well), and incubated for 1 hour at 37°C in 5% CO_2_. At the end of incubation, the MDCK cell suspension (1.5 × 10^5^ cells/mL) was added to the plates, which were then stored in an incubator (37°C, 5% CO_2_) for 5 days. After incubation, the plates were observed by optical microscopy and evaluated for cytopathic effect. A cytopathic effect higher than 50% indicates infection. The titer was expressed as the inverse of the last dilution that showed inhibition of cytopathic effect.

### 2.6 ELISA

To evaluate humoral responses, the ELISA was performed on human serum samples in 96‐well flat bottom half‐area Microlon titer plates (Greiner, Alphen a/d Rijn, The Netherlands). Antigens used for ELISA coating were lyophilized SRID antigen, obtained from NIBSC (Potters Bar, Hertfordshire, United Kingdom). Plates were coated overnight at 4°C with 1 μg/mL hemagglutinin (50 μL/well in PBS) of the relevant influenza antigens.

Following blocking with 100 μL/well of 3% BSA (Sigma, Zwijndrecht, The Netherlands) in PBS‐T, plates were washed and samples were loaded. Human serum samples were loaded onto the plates and incubated for 1 hour at room temperature. Samples were tested at a starting dilution of 1:500 (H1N1, H3N2, and B/Yamagata lineage) or 1:1000 (B/Victoria lineage) and 3‐fold serially diluted over four wells. A pool of human sera diluted 2‐fold over seven wells was used as a calibrator on every plate. After incubation, plates were washed and incubated for 1 hour at room temperature with 50 μL/well of 1:1250 diluted goat anti‐human IgG conjugated to alkaline phosphatase (Thermo Fisher Scientific, Etten‐Leur, The Netherlands). ELISA was developed with 50 μL/well p‐nitrophenyl phosphate (pNPP; Fluka, Poole, UK) for 30 minutes. The optical density (OD) was read at 405 nm using a Bio‐Rad plate reader (model iMark—microplate reader). ODs were converted to arbitrary units (AUs) using a four‐parameter logistic fit (ADAMSEL, http://www.malariaresearch.eu), where 1 AU yields an OD of 1 over the background. Thus, the amount of AU of a sample is the reciprocal dilution at which an OD of 1 over the background is achieved.

### 2.7 Statistical analysis

All statistical analyses were performed using Microsoft R‐Open, version 3.4.3 (R Core Team (2018); R: A language and environment for statistical computing; R Foundation for Statistical Computing, Vienna, Austria. Figures were prepared using the ggplot2 package in Microsoft R‐open. Correlation (Pearson product‐moment correlation coefficient, Pearson's *r*) and linear regression analyses were performed on log 2 transformed data for HI, MN, and ELISA. SRH data were not log transformed. Kappa statistics were calculated using the R package fmsb (https://CRAN.R-project.org/package=fmsb).

## 3 RESULTS

The human serum samples (n = 450) were tested by HI, SRH, MN, and ELISA assays to evaluate the assay correlation using four seasonal influenza strains: A/California/7/2009 (H1N1), A/Texas/50/2012 (H3N2), B/Brisbane/60/2008 (Victoria lineage), and B/Massachusetts/02/2012 (Yamagata lineage).

**Figure 1:**
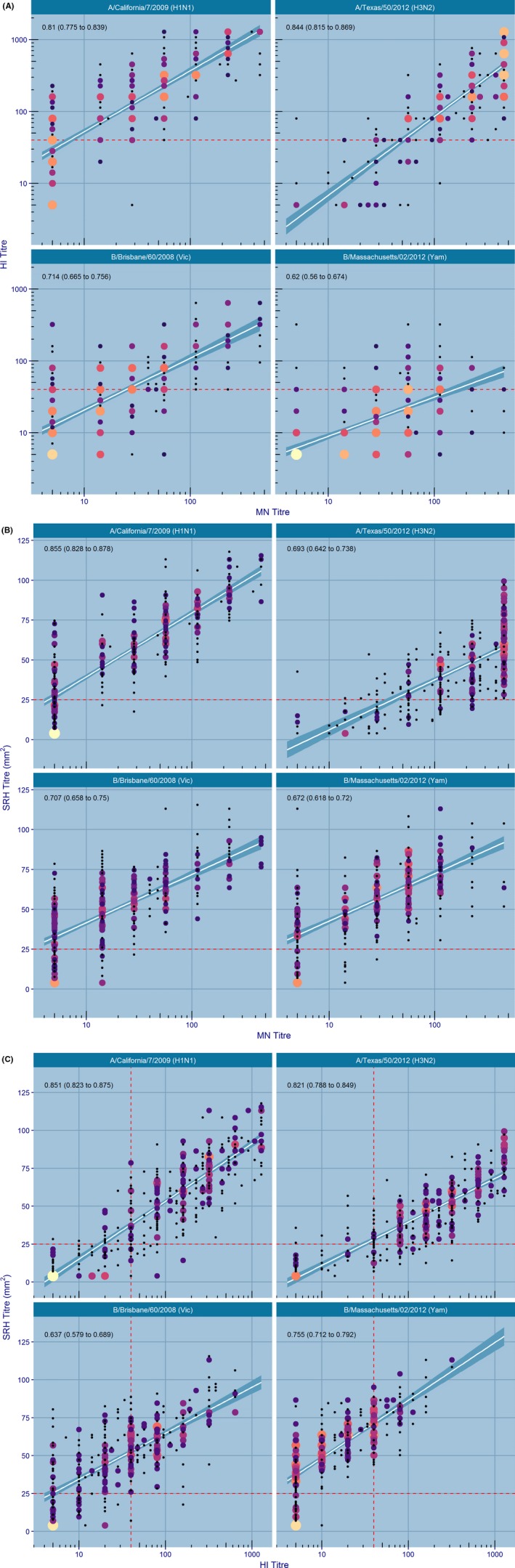
Correlation between HI‐MN, HI‐SRH, and SRH‐MN titers. Size and color indicate the number of observations at the same coordinates. The size of the circle is proportional to the square root of the number of observations at that position; thus, the size is directly proportional to the number of observations. Symbol colors also indicate the number of observations (magma color), where black indicates single observations and yellow indicates many observations. A, 
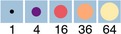
. B, 
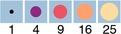
. C,
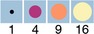

For the influenza A strains, a strong positive correlation was found between HI and MN assays (Pearson's *r* = 0.81‐0.84), SRH and HI assays (Pearson's *r* = 0.82‐0.85), and SRH and MN assays (Pearson's *r* = 0.69‐0.86) (Figure 1; Table 1).

**Table 1:** Correlation coefficients and regression estimates

Strain	Comparison	*X* value	*Y* value	Slope	Intercept	Correlation coefficient (*r* 95% CI)
A/California/7/2009 (H1N1)	HI ~ MN	MN	HI	0.849	2.895	0.810 (0.775‐0.839)
A/Texas/50/2012 (H3N2)	HI ~ MN	MN	HI	1.095	−0.865	0.844 (0.815‐0.869)
B/Brisbane/60/2008 (Vic)	HI ~ MN	MN	HI	0.721	1.977	0.714 (0.665‐0.756)
B/Massachusetts/02/2012 (Yam)	HI ~ MN	MN	HI	0.544	1.356	0.620 (0.560‐0.674)
A/California/7/2009 (H1N1)	SRH ~ MN	MN	SRH	12.074	−0.657	0.855 (0.828‐0.878)
A/Texas/50/2012 (H3N2)	SRH ~ MN	MN	SRH	9.446	−25.153	0.693 (0.642‐0.738)
B/Brisbane/60/2008 (Vic)	SRH ~ MN	MN	SRH	9.102	11.411	0.707 (0.658‐0.750)
B/Massachusetts/02/2012 (Yam)	SRH ~ MN	MN	SRH	8.953	12.958	0.672 (0.618‐0.720)
A/California/7/2009 (H1N1)	SRH ~ HI	HI	SRH	11.465	−22.954	0.851 (0.823‐0.875)
A/Texas/50/2012 (H3N2)	SRH ~ HI	HI	SRH	8.620	−17.660	0.821 (0.788‐0.849)
B/Brisbane/60/2008 (Vic)	SRH ~ HI	HI	SRH	9.132	4.108	0.637 (0.579‐0.689)
B/Massachusetts/02/2012 (Yam)	SRH ~ HI	HI	SRH	11.456	10.424	0.755 (0.712‐0.792)
A/California/7/2009 (H1N1)	HI ~ ELISA	IgG	HI	0.905	−4.227	0.613 (0.552‐0.668)
A/Texas/50/2012 (H3N2)	HI ~ ELISA	IgG	HI	0.991	−4.708	0.664 (0.609‐0.713)
B/Brisbane/60/2008 (Vic)	HI ~ ELISA	IgG	HI	0.773	−5.463	0.544 (0.476‐0.606)
B/Massachusetts/02/2012 (Yam)	HI ~ ELISA	IgG	HI	0.588	−3.371	0.604 (0.542‐0.660)
A/California/7/2009 (H1N1)	MN ~ ELISA	IgG	MN	0.867	−5.950	0.615 (0.554‐0.670)
A/Texas/50/2012 (H3N2)	MN ~ ELISA	IgG	MN	0.676	−0.733	0.589 (0.525‐0.646)
B/Brisbane/60/2008 (Vic)	MN ~ ELISA	IgG	MN	0.848	−7.281	0.604 (0.542‐0.660)
B/Massachusetts/02/2012 (Yam)	MN ~ ELISA	IgG	MN	0.571	−2.330	0.514 (0.443‐0.579)
A/California/7/2009 (H1N1)	SRH ~ ELISA	IgG	SRH	13.556	−110.250	0.681 (0.629‐0.728)
A/Texas/50/2012 (H3N2)	SRH ~ ELISA	IgG	SRH	11.148	−89.966	0.712 (0.663‐0.755)
B/Brisbane/60/2008 (Vic)	SRH ~ ELISA	IgG	SRH	11.519	−106.559	0.637 (0.579‐0.689)
B/Massachusetts/02/2012 (Yam)	SRH ~ ELISA	IgG	SRH	10.622	−76.565	0.719 (0.671‐0.761)

Correlation coefficients (Pearson's *r*) and regression estimates for slope and intercept. HI, MN, and ELISA were log 2 transformed; SRH titer was used without transformation.

Strong positive correlations were also found between HI and MN (Pearson's *r* = 0.62‐0.71), SRH and HI (Pearson's *r* = 0.64‐0.75), and SRH and MN assays (Pearson's *r* = 0.67‐0.71) for B/Massachusetts/02/2012 and B/Brisbane/60/2008, respectively. Notably, correlations for the B/Massachusetts/02/2012 were consistently lower than those for B/Brisbane/60/2006.

**Figure 2:**
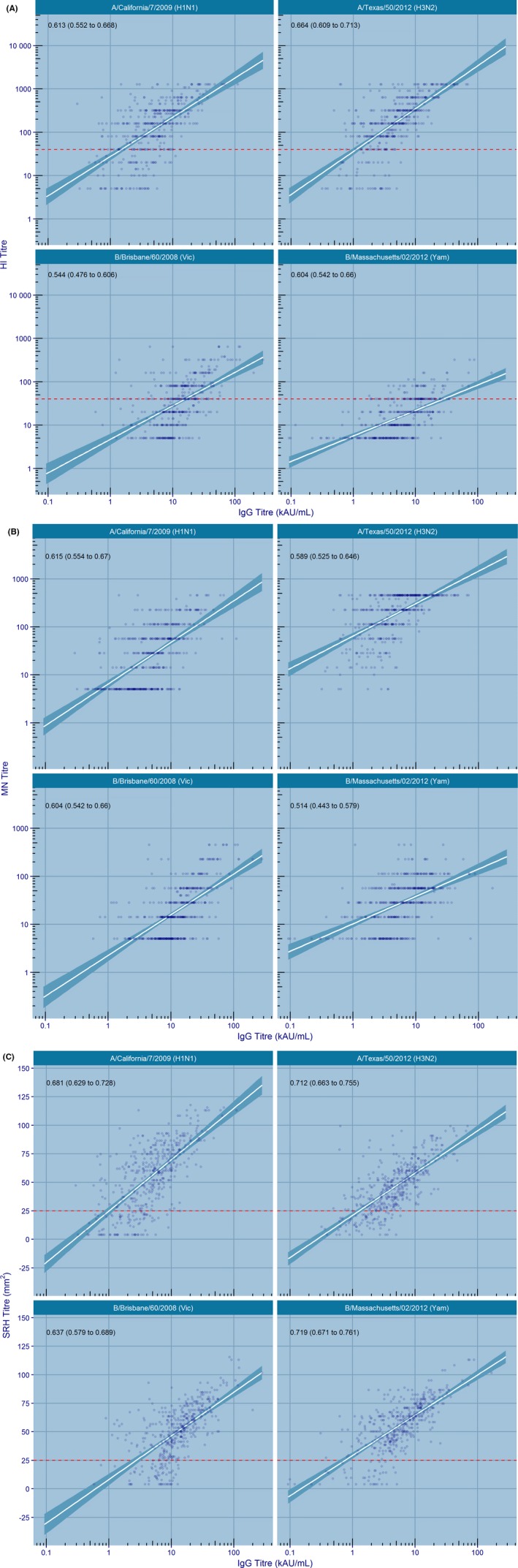
Correlation between ELISA (IgG titer)‐HI, ELISA (IgG titer)‐MN, and ELISA (IgG titer)‐SRH titers. The points are plotted in such a way as to show where the majority of observations are located

Positive correlations were also found between ELISA and HI assays (0.61 A/California/07/2009, 0.66 A/Texas/50/2012, 0.54 B/Brisbane/60/2009, 0.60 B/Massachusetts/02/2012), ELISA and MN assays (0.61 A/California/07/2009, 0.59 A/Texas/50/2012, 0.60 B/Brisbane/60/2009, 0.51 B/Massachusetts/02/2012), and ELISA and SRH assays (0.68 A/California/07/2009, 0.71 A/Texas/50/2012, 0.64 B/Brisbane/60/2009, 0.72 B/Massachusetts/02/2012) (Figure 2; Table 1).

For the A strains, the correlation between HI, MN, and SRH was stronger than that between ELISA and the other assays. By contrast, the correlation between ELISA and HI, and SRH and VN assays was comparable to that of the three traditional assays for the B strains.

In addition, the assay agreement on protection, as defined by HI ≥ 40 and SRH ≥ 25 mm^2^, was evaluated using Cohen's kappa statistic. The kappa statistic measures inter‐rater agreement for categorical variables while correcting for chance. The kappa statistics showed good agreement for the A/California/07/2009 (H1N1) (*k* = 0.72) and A/Texas/50/2012 (H3N2) (*k* = 0.59) influenza strains, indicating that most subjects were considered to be protected on the basis of both HI and SRH threshold levels. By contrast, the correlation for the B/Brisbane/60/2008 (*k* = 0.34) and B/Massachusetts/02/2012 (*k* = 0.09) strains was very low, especially for the latter. These data suggest that a high number of subjects are considered to be protected on the basis of the SRH protective threshold level but not the HI threshold. When kappa statistics were repeated, assuming an HI threshold of 20 for the B strains, kappa values were 0.53 for B/Brisbane/60/2008 and 0.18 for B/Massachusetts/02/2012.

## 4 DISCUSSION

The immunological response to influenza natural infection or vaccination is usually evaluated by serological assays such as HI, SRH, and MN. The HI assay is considered the gold standard as a correlate of protection for influenza vaccine and detects antibodies able to bind the viral HA and inhibit virus‐red blood cells agglutination. The SRH assay recognizes complement activating antibodies, while the MN assay identifies functional neutralizing antibodies able to prevent the entry or replication of the virus in mammalian cells. All three assays are officially recognized by the EMA for the evaluation of influenza vaccine immunogenicity.^1^

The aim of the study was to compare the HI, SRH, MN assays, and ELISA using four seasonal influenza strains (A/California/7/2009 (H1N1), A/Texas/50/2012 (H3N2), B/Brisbane/60/2008 Victoria lineage, and B/Massachusetts/02/2012 Yamagata lineage).

The data highlight strong correlations between HI, MN, and SRH assays for influenza A strains (H1N1 and H3N2). A significant correlation between HI and MN has been found by Veguilla *et al* 27 with the same H1N1 strain used in this study, and, in other studies, with different influenza strains, such as A/Brisbane/10/2007 (H3N2), A/Brisbane/59/2007 (H1N1), and equine influenza viruses.^11^, ^29^, ^30^, ^33^ Previous studies involving A strains support the strong agreement between HI and SRH assays found in present study.^11^, ^14^, ^28^ The correlation between SRH and MN assays observed in this study is in agreement with the findings of the previous studies that used equine influenza strains and H3N2 influenza strain.^11^, ^29^, ^30^ However, unlike Wang et al*,* 30 who found much higher correlations between SRH and MN assays than between SRH‐HI and HI‐MN assays, we found no significant differences among them.

The correlation among the three assays was also evaluated for influenza B strains (Victoria and Yamagata lineages), showing good agreement. These values were slightly lower than for the A strains; however, the difference was small. This could be due to the poor sensitivity of the HI assay to B strains. We found only one previous study of B strains, but this was limited to HI and SRH, which were applied only to the B/Beijing/1/87 strain; this found a statistically significant correlation between the two assays supporting our data.^14^

Although ELISA is not officially recognized by the EMA, the technique is able to measure different class‐specific IgM, IgA, and IgG antibodies. In addition, the assay is able to measure HA stalk‐specific antibodies and could be used to evaluate the immunogenicity of novel universal influenza vaccines.^34^ ELISA is particularly suitable for large‐scale serological investigation, as it yields unbiased results in a few hours and allows complete automation of the process by high‐throughput testing. Our data reveal a positive correlation between ELISA and the other three assays, with lower values for A strains but without differences between A and B strains. Only two previous studies have evaluated the correlations among ELISA, MN, and HI, and, in agreement with our data, have shown good correlations among them.^31^, ^32^ The present study seems to be the first to evaluate the immunological correlation between ELISA and SRH assay.

Until February 2017, the correlates of protection had been established for the HI and SRH assays only. Traditionally, an HI titer ≥ 40 was considered an immunological correlate of protection and the best available parameter for predicting protection from influenza infection; a hemolysis area of 25 mm^2^ or greater was generally regarded as a protective threshold level, beyond which the probability of contracting influenza infection was reduced by 50% or more.^7^, ^35^ Although the traditional correlates of protection have been used for decades, they have been questioned. First of all, the correlates of protection only targeted healthy adults aged 18‐60 years and the over‐60s, thereby excluding children and other subjects at high risk. The study by Black *et al* 36 demonstrated that it is inappropriate to use an HI titer ≥ 40 as a correlate of protection for children under 6 years of age, as they need an HI titer of 110 to reach a 50% protection level. In addition, there was no difference between adjuvanted and non‐adjuvanted vaccines, no defined correlates of protection specific to live attenuated influenza and pandemic vaccines, and the use of the HI assay itself has been questioned.^8^, ^9^, ^36^, ^37^, ^38^ However, the new EMA guidelines^1^ have withdrawn the concept of the “traditional correlates of protection,” meaning that an HI titer ≥ 40 and a hemolysis area of 25 mm^2^ or greater are no longer accepted as a threshold of seroprotection.

The data show that there is good agreement between both assays with regard to protection against the A strains. This means that subjects who are considered to be protected on the basis of HI threshold levels are also deemed to be protected on the basis of SRH threshold levels. By contrast, our comparison of correlates of protection for the B strains showed low and very low agreement, particularly for the Massachusetts strain. Unfortunately, we found few previous studies that compare the correlates of protection of HI and SRH assays. Nevertheless, these studies support our data and reveal that SRH is more sensitive than HI in detecting antibodies against influenza B viruses.^39^, ^40^ If the threshold level applied is reduced from 40 to 20, the level of agreement increases slightly, suggesting that the protective HI level for B strains may be lower, as suggested by Hobson&Curry.^2^ To increase the sensitivity of the assay, the WHO requires that influenza B viruses be ether‐treated for the serological diagnosis of influenza B infection; however, this treatment could reduce the specificity of the assay.^41^, ^42^, ^43^

The present study has some limitations, such as missing information on the vaccination status of the subjects involved and the unavailability of paired serum samples. Another limitation is the lack of comparability between ether‐treated B viruses and native viruses and between egg‐grown and cell‐grown viruses.

Overall, this study shows a strong positive correlation among the four serological assays (MN, HI, SRH, and ELISA) for both A and B strains; this is especially true of the HI and MN assays. However, it also highlights the need to further investigate the correlation between the SRH assay and ELISA. Concerning the correlates of protection, as defined by HI ≥ 40 and SRH ≥ 25 mm^2^, we found good agreement regarding protection against A strains between HI and SRH assays, but very low agreement for the B strains, suggesting that SRH is more sensitive than HI in detecting antibodies against the influenza B viruses. As the four serological assays detect different sets or subsets of antibodies, combining all the assays could considerably improve the assessment of the immunogenicity of influenza vaccines and provide a more complete picture of antibody responses. In addition, it could be useful to establish the correlates of protection for the MN assay, in order to compare vaccine assessments based on the three assays. Finally, further research on ELISA could be particularly useful in order to evaluate the immunogenicity of novel universal influenza vaccines.

## CONFLICT OF INTERESTS

The authors have no competing interests.

## AUTHORS' CONTRIBUTION

E.M. conceived the study; E.M. and C.M.T. designed the experiments; C.M.T. and D.M. performed the experiments; E.J.R. analyzed the data; C.M.T. drafted the study; E.J.R. revised the study. All the authors revised and approved the final version of the manuscript.
